# D-SNP Enrollee Healthcare Access and Satisfaction: Does Insurance Level of Integration Make a Difference?

**DOI:** 10.1093/geroni/igaf122.3336

**Published:** 2025-12-31

**Authors:** Morgan Perry, Ishrat Alam, Geoffrey Hoffman

**Affiliations:** University of Michigan, Ann Arbor, Michigan, United States; University of Michigan, Ann Arbor, Michigan, United States; University of Michigan, Ann Arbor, Michigan, United States

## Abstract

Dual Eligible Special Needs Plans (D-SNPs) range from less integrated, coordination-only plans (CO D-SNPs) with enhanced communication between Medicare Advantage and Medicaid to fully integrated plans (FIDE-SNPs) managed under a single entity. Using restricted Medicare Current Beneficiary Survey data (2015-2020) and CMS health plan data, we assessed the relationship between enrollee satisfaction and access with health plan integration. We identified community-dwelling, fully dually eligible individuals enrolled in a D-SNP, resulting in 3,369 CO D-SNP and 341 FIDE-SNP enrollee-year observations. We estimated survey-weighted logistic regression models adjusting for state and year fixed effects plus individual and area-level factors. We conducted subgroup analyses by age and race/ethnicity. Self-reported satisfaction with access and quality of care were generally high. While FIDE-SNP enrollees were more likely to recommend Medicare Advantage (Adjusted Risk Ratio [ARR]: 1.04, p = 0.006), other outcomes did not vary by level of integration. Comparing FIDE to CO plans, enrollees under 65 were more likely to report having a regular source of care (ARR: 1.07, p = 0.019), those 65 and older were more likely to recommend Medicare Advantage (ARR: 1.05, p = 0.003) and report being satisfied with specialty care (ARR 1.04; p = 0.017), and White enrollees were more likely to report being very satisfied with specialty care (ARR: 1.56, p = 0.003) and plan-related costs (ARR: 1.42, p = 0.002). Mixed sub-group findings call into question whether recent investments into FIDE-SNPs are justified and equitable across populations. State policymakers should consider more tailored investments while awaiting more findings on the effectiveness and equity of these approaches.

